# Erythema Scarlatiniforme Desquamativum Recidivans: A Rare and Puzzling Condition

**DOI:** 10.7759/cureus.68981

**Published:** 2024-09-09

**Authors:** Ghita Benbrahim Ansari, Houda Naji, Hanan Aboufaris, Kenza Bouayed

**Affiliations:** 1 Pediatric Rheumatology, Abderrahim Harouchi Mother and Child Hospital, Centre Hospitalier Universitaire (CHU) Ibn Rochd, Casablanca, MAR; 2 Pediatric Rheumatology, Faculty of Medicine and Pharmacy, Hassan 2 University, Casablanca, MAR

**Keywords:** prodromal phase, uncommon condition, children, desquamation, recurrent scarlatiniforme rash

## Abstract

Recurrent erythema scarlatiniforme desquamativum recidivans (ESDR), also known as Féréol-Besnier disease, is a rare condition marked by a recurrent erythematous rash that is followed by extensive desquamation, primarily affecting the palms and soles. It is often preceded by prodromal symptoms such as malaise, headache, myalgias, digestive issues, and fever. The exact pathogenesis remains unknown, and diagnosis can be challenging due to its resemblance to various infectious, auto-inflammatory, or allergic conditions, leading to diagnostic variability. Given that most reported cases are over 50 years old, our objective is to highlight this rare and enigmatic pathology through a typical case of the generalized variant of ESDR in a 13-year-old girl. We aim to increase physician awareness of this condition and provide reassurance to both parents and their child regarding its benign nature.

## Introduction

Erythema scarlatiniforme desquamativum recidivans (ESDR) is a rare condition marked by recurrent episodes of erythema followed by extensive lamellar scaling, particularly affecting the palms and soles [1,2]. The rash is preceded by a prodromal phase that includes general malaise, headache, arthralgia, myalgia, digestive symptoms, and fever [3]. ESDR is characterized by episodes of cutaneous flare-ups that resolve spontaneously, with intervals of variable duration ranging from a few weeks to several months or even years. The condition has two variants: generalized and localized [2]. To illustrate this uncommon skin disorder, we present a typical case of the generalized variant of ESDR in a 13-year-old girl.

## Case presentation

Our patient was a healthy 13-year-old girl with a history of recurrent episodes of generalized pruritic macular erythema followed by lamellar skin peeling that regressed spontaneously. These episodes were preceded by fever, joint and muscle pain, asthenia, and headache. She had no history of atopy, drug hypersensitivity, familial cases, or other triggering factors, including medication or infections. The first episode occurred seven years ago. Initially, the symptoms recurred monthly for four years. Over the last three years, she experienced an average of three eruptions per year, each lasting about two weeks and resolving without treatment.

**Figure 1:**
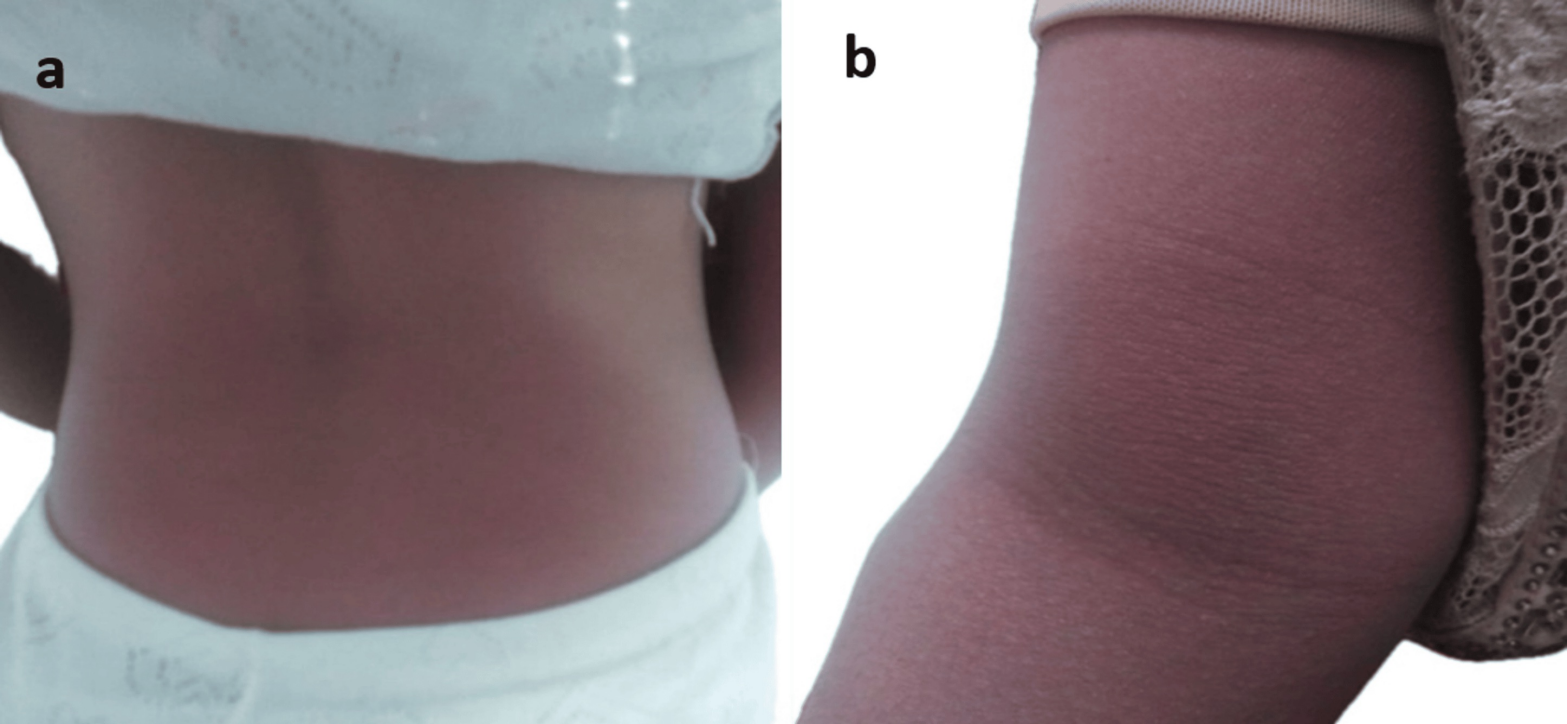
(a, b) Diffuse macular skin rash on the trunk and limbs

She presented to our facility with a recent rash. Physical examination revealed diffuse macular lesions on the trunk, neck, and limbs (Figure 1), which extended to the hands and feet. The face and mucous membranes were spared. The patient was in good general condition and had no fever. The remainder of the clinical examination was normal.

**Figure 2:**
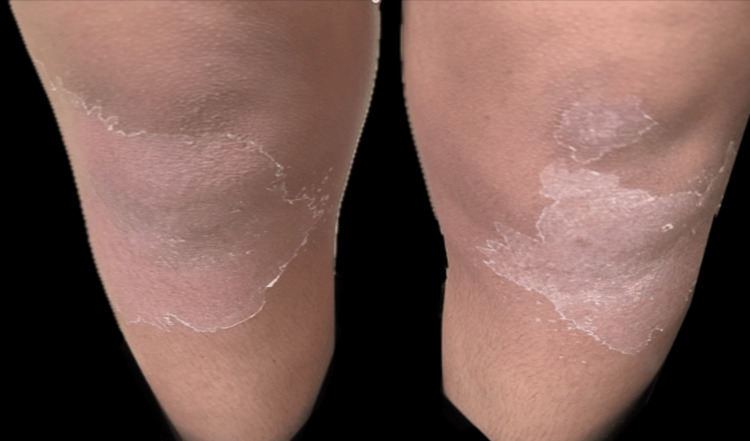
Lamellar desquamation on the limbs

**Figure 3:**
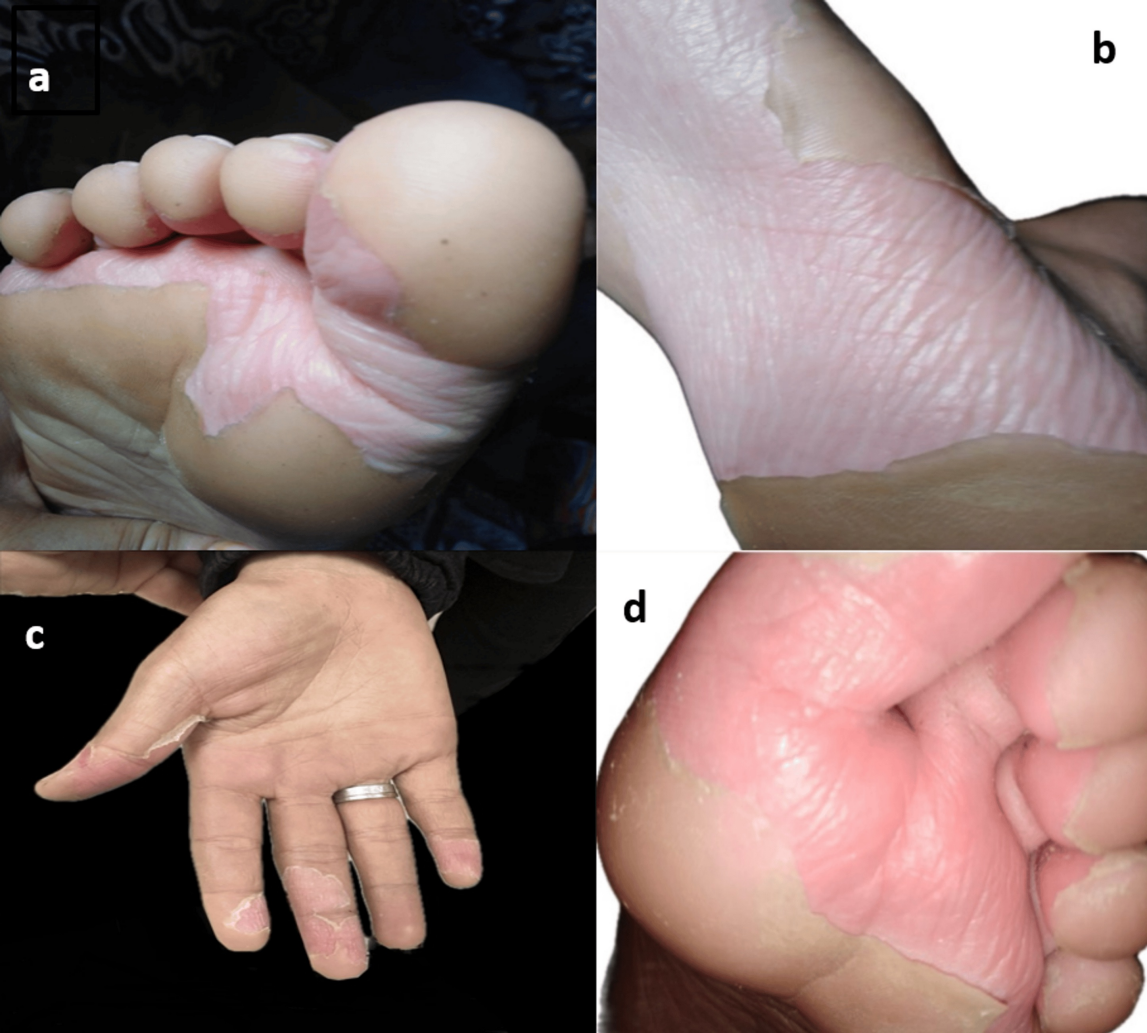
(a, b, c) Desquamation with hyperkeratotic thickened skin on the foot and hand. (d) Post-desquamation: soft and erythematous skin

One week after the onset of the skin eruption, significant lamellar desquamation was observed on the trunk and limbs (Figure 2), along with thickened, ragged peeling of the palmar and plantar skin (Figure 3). Laboratory tests showed a mildly elevated CRP level of 16 mg/L, a normal leukocyte count of 8,080/mm³ (with neutrophils at 2,070/mm³ and eosinophils at 102/mm³), an erythrocyte sedimentation rate (ESR) of 4 mm/1st hour, and a normal anti-streptolysin O level of 145 U/L (Table 1). Histological examination of the skin revealed epidermal hyperplasia with hyperorthokeratosis. The dermis showed congested vessels and perivascular lymphocytic infiltration. There were no apoptotic keratinocytes or eosinophilic infiltration; this aspecific histological appearance did not contribute to the diagnosis. Following topical therapy with emollients and keratolytics, the lesions resolved within two weeks.

**Table 1:** Summary of laboratory findings for the presented case

Laboratory findings	Values
CRP	16 mg/l (<6 mg/L)
Leukocyte count	8,080/mm³ (5,000-10,000/mm³)
Neutrophils	2,070/mm³ (1,500-7,000/mm³)
Eosinophils	102/mm³ (50-500/mm³)
Erythrocyte sedimentation rate	4 mm/1st hour (<10 mm/1st hour)
Anti-streptolysin O	145 UI/L (<200 UI/L)

## Discussion

ESDR is a rare disorder characterized by recurrent erythematous rashes followed by extensive desquamation, primarily affecting the palmoplantar skin [4,5]. First described by Féréol in 1876 [6], only a few cases have been documented since (Table 2) [1,3,5-9]. The rarity of ESDR is likely due to limited awareness [3,5], and the scarcity of literature results in a lack of reliable epidemiological data on its incidence and prevalence [2].

**Table 2:** Reported cases of ESDR ASLO: antistreptolysin O antibodies; ESDR, erythema scarlatiniforme desquamans; ESR: erythrocyte sedimentation rate

	Age (year)	Sex	ESDR variant	Trigger factor	Clinical/laboratory findings	References
Patient 1	40	male	Generalized ESRD	none	Fever, headache, throat pain, recurrent aseptic meningitis, normal value of ASLO, and slight transient proteinuria	7
Patient 2	56	Female	Localized ESDR	Drug-induced salicylates and diuretics	Increased value of ASLO	8
Patient 3	54	Male	Localized ESDR	Staphylococcal infection	ESR: 45mm/1st hour and WBC: 13,000/mm³; Staphylococcus aureus isolated from tonsillar smear	8
Patient 4	86	Female	Generalized ESRD	None	Fever CRP: 27.6 mg/l, WBC: 11,820/mm³, and neutrophils: 92.8%	6
Patient 5	22	Female	Localized ESDR	None	No prodromal phase, normal WBC, ESR: 38 mm/1st hour, and normal flora on the bacterial culture of the pharyngeal cavity	1
Patient 6	66	Male	Localized ESDR	None	General malaise, normal laboratory findings, and normal swab of the pharyngeal cavity	1
Patient 7	65	Female	Localized ESDR	Dental surgery six weeks before the first episode	No prodromal phase, normal ASLO titer, and bacterial culture of the pharynx	1
Patient 8	54	Male	Localized ESDR	Acetylsalicylic acid, fluvastatin, and bisoprolol intake; exposure to metallic vapor	No prodromal phase, normal white blood cells, elevated CRP: 8.8 mg/dl, normal screening for viral infections, normal ASLO titer, and normal flora on bacterial culture of the pharyngeal and nasal cavities	3
Patient 9	57	Male	Localized ESDR	Infection of the upper respiratory tract in two episodes	Back pain, burning sensation on the tongue, dysgeusia, mild swelling of the lips, C-glutamyl transpeptidase: 197 U/L (normal <66 U/L); CRP: 4.5 mg ⁄dl, and normal flora on the bacterial culture of the pharyngeal cavity	5
Patient 10	22	Male	Localized ESDR	Nasopharyngitis	Fever, throat pain, increased levels of inflammatory parameters, and negative rapid strep test	9
Our patient	13	Female	Generalized ESRD	None	Fever, joint pain, asthenia, headache, muscle aches, ESR: 4 mm/1^st^ hour, WBC: 8,080/mm³, eosinophils: 102/mm³, and CRP: 16 mg/l	

ESDR presents in two forms: a generalized variant, which appears to be more common, and a localized variant [3,6]. The generalized form, which our case exemplifies, features confluent macular erythema initially on the trunk, spreading to the limbs and face, followed by lamellar desquamation [1-3]. Pruritus may also be observed, as in our patient, while mucous membranes are typically unaffected [2]. The rash is preceded by a prodromal phase with general malaise, headaches, muscle aches, gastrointestinal symptoms, fever, joint pain, or bronchitis [1,3,5]. Additional clinical features may include conjunctivitis, pharyngitis, epistaxis, alopecia, transient proteinuria, and microhematuria [1,3]. The condition is marked by acute episodes lasting two to three weeks, resolving spontaneously, with asymptomatic intervals between episodes ranging from months to years [5,6].

The etiology and pathogenesis of ESDR remain unknown [1]. Triggering factors reported include drug hypersensitivity, viral and bacterial infections, particularly staphylococcal and streptococcal [2]. In our patient, no specific etiology was identified.

Diagnosis is primarily clinical, as histological and laboratory findings are nonspecific [1]. Blood tests typically reveal elevated inflammation markers, such as an increased erythrocyte sedimentation rate or CRP, and sometimes hyperleukocytosis, with occasional hypereosinophilia [3]. In our patient, inflammatory parameters were normal except for a slightly elevated CRP. Histological examination may show hyperparakeratosis, orthokeratosis, acanthosis above the stratum granulosum, and inflammation [2,3]. Differential diagnoses include scarlet fever, staphylococcal skin infections, toxic epidermal necrolysis, and recurrent fevers like familial Mediterranean fever or mevalonate kinase deficiency [3].

Currently, there are no specific guidelines for treating this rare benign condition. Symptomatic treatments, including topical and systemic steroids, systemic antibiotics, emollients, and topical keratolytics, may be useful but do not alter the natural course of the disease or prevent new episodes [1,3,5].

## Conclusions

Although well-documented, ESDR remains a rare and often underdiagnosed skin condition. It should be considered in any patient presenting with recurrent erythema followed by lamellar desquamation, once other differential diagnoses have been excluded. Recognizing ESDR can help reassure parents regarding the prognosis. Symptomatic therapy is currently the primary approach for managing ESDR. Ongoing research and case studies are essential for refining diagnostic criteria and therapeutic strategies to better address this condition.
